# Structure, regulation, and physiological functions of NADPH oxidase 5 (NOX5)

**DOI:** 10.1007/s13105-023-00955-3

**Published:** 2023-03-11

**Authors:** Jorge G. García, Eduardo Ansorena, Iñigo Izal, Guillermo Zalba, Carlos de Miguel, Fermín I. Milagro

**Affiliations:** 1 Department of Biochemistry and Genetics, University of Navarra, 31008 Pamplona, Spain; 2 Navarra Institute for Health Research (IdiSNA), 31008 Pamplona, Spain; 3 Department of Nutrition, Food Science, and Physiology and Center for Nutrition Research, University of Navarra, 31008 Pamplona, Spain; 4 Centro de Investigación Biomédica en Red Fisiopatología de La Obesidad Y Nutrición (CIBERobn), Instituto de Salud Carlos III, Madrid, Spain

**Keywords:** NADPH oxidase, NOX, Insulin resistance, Obesity, Oxidative stress, Inflammation

## Abstract

NOX5 is the last member of the NADPH oxidase (NOXs) family to be identified and presents some specific characteristics differing from the rest of the NOXs. It contains four Ca^2+^ binding domains at the N-terminus and its activity is regulated by the intracellular concentration of Ca^2+^. NOX5 generates superoxide (O_2_^•−^) using NADPH as a substrate, and it modulates functions related to processes in which reactive oxygen species (ROS) are involved. Those functions appear to be detrimental or beneficial depending on the level of ROS produced. For example, the increase in NOX5 activity is related to the development of various oxidative stress-related pathologies such as cancer, cardiovascular, and renal diseases. In this context, pancreatic expression of NOX5 can negatively alter insulin action in high-fat diet-fed transgenic mice. This is consistent with the idea that the expression of NOX5 tends to increase in response to a stimulus or a stressful situation, generally causing a worsening of the pathology. On the other hand, it has also been suggested that it might have a positive role in preparing the body for metabolic stress, for example, by inducing a protective adipose tissue adaptation to the excess of nutrients supplied by a high-fat diet. In this line, its endothelial overexpression can delay lipid accumulation and insulin resistance development in obese transgenic mice by inducing the secretion of IL-6 followed by the expression of thermogenic and lipolytic genes. However, as *NOX5* gene is not present in rodents and human NOX5 protein has not been crystallized, its function is still poorly characterized and further extensive research is required.

## NADPH oxidases 

**Fig. 1:**
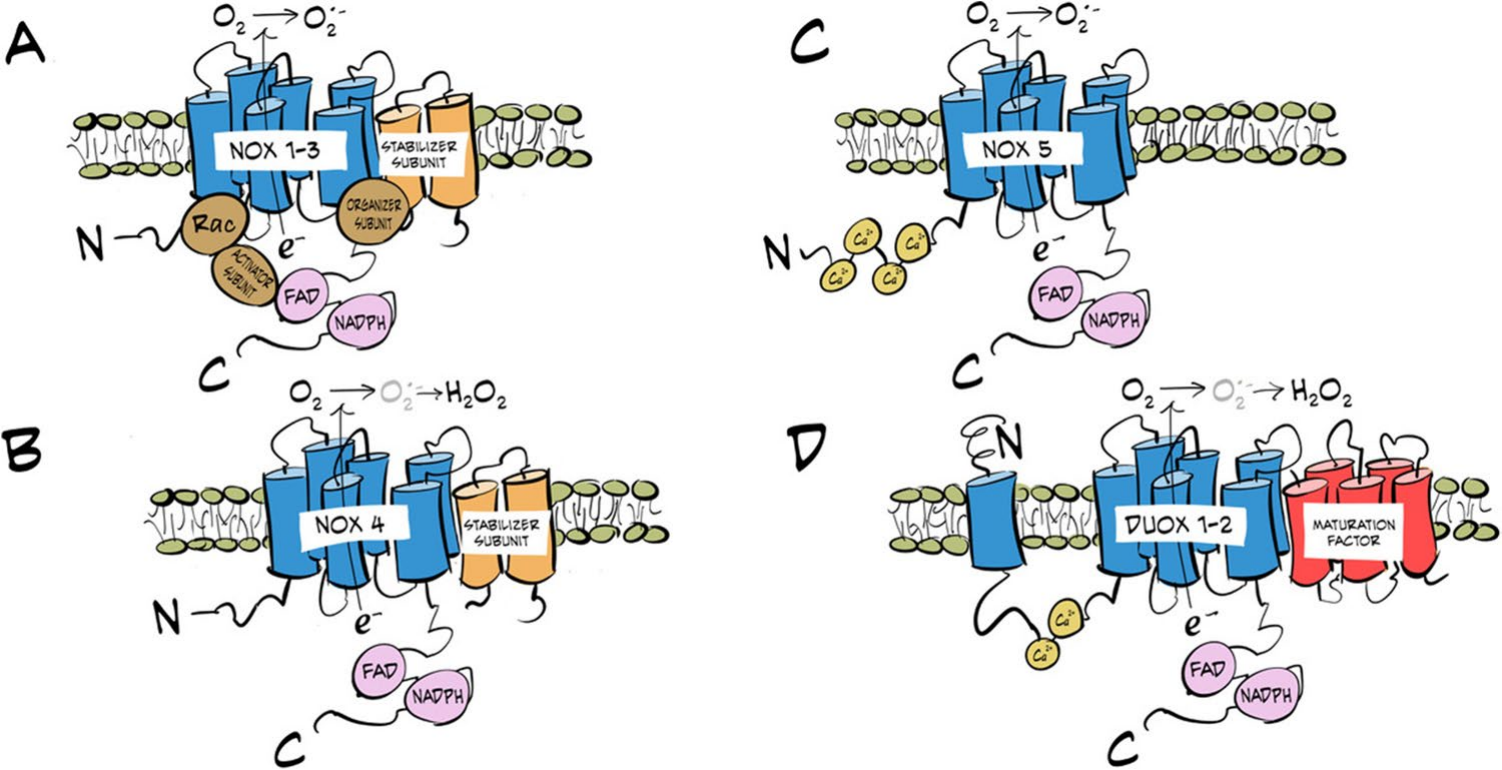
Schematic representation of the structure of the different NOXs. **A** Structure of isoforms 1–3. **B** Structure of isoform 4. **C** Structure of isoform 5. **D** Structure of DUOX1-2 isoforms. The transmembrane domains (blue), the subunits necessary for the activation of each enzyme (brown), the stabilizer subunits (orange), and maturation factors in DUOX (red) and the FAD/NADH binding domains (purple) are indicated in each figure. Image adapted from Buvelot et al. [17]

The NADPH oxidase (NOXs) family is characterized for being membrane proteins that use NADPH as an electron donor. The first enzyme belonging to this family of oxidases was identified in 1986. It was located in the membrane of phagocytic cells and it was found to be a protein capable of generating H_2_O_2_ using NADPH as a substrate. This enzyme was named NOX2/gp91PHOX. Since then, six more isoforms have been identified, constituting the family of NADPH oxidases (Fig. 1), whose 7 members are called NOX 1–5 and dual oxidases DUOX 1–2 [15, 17, 94]. As explained below, the DUOX family constitutes a different subfamily due to their extended primary sequence, the type of ROS formed, their maturation process, and their functional regulation [29]. The NOX members share the same catalytic core: a six-transmembrane helical domain (TM) with a C-terminal cytosolic dehydrogenase domain (DH). The DH domain includes the binding sites for FAD (flavin adenine dinucleotide) and NADPH, whereas TM binds two hemes [15, 73].

NOX2 is the most studied member of those oxidases. It can be found in cardiomyocytes, myocytes, hepatocytes, and endothelial cells. Its gp91phox subunit, also cataloged as NOX2, represents the catalytic center. The p22phox, p47phox, and p67phox subunits form the stabilizing, organizing, and activating subunits, respectively [44, 46, 49, 55, 92]. The mechanism by which this enzyme and the rest of NOXs generates O^2•−^ is the following: NADPH binds to FAD and transfers two electrons to it. As a result, FAD is reduced to FADH2 and transfers an electron to the heme group located on the gp91phox subunit. Consecutively, the latter will reduce the O_2_ molecule to produce O_2_^•−^. FADH^•^ can repeat the process faster generating another molecule of O_2_^•−^ [28, 46].

The NOX1 isoform has 60% sequence identity to NOX2. It is expressed in various tissues such as the epithelium of the colon, uterus, prostate, and muscle and in endothelial cells. Like NOX2, NOX1 acts as a catalytic center for the generation of O_2_^•−^; p22phox is the stabilizing subunit; NOXO1 is the organizer; and NOXO1A is the activator [2, 10, 99]. In the case of NOX3, its sequence identity to NOX2 is 56%. Like NOX1, this enzyme complex is composed of NOX3, p22phox, NOXO1, and NOXO1A. It is expressed in tissues such as the inner ear and its main characteristic is that it can be activated without the need for an external stimulus [11, 104].

Interestingly, NOX4 protein is not as similar to NOX2 as the previous isoforms. Its distribution is especially abundant in the kidney, but it is also expressed in endothelium and muscle. The enzymatic complex of this protein is formed only by two proteins: NOX4, which is the catalytic center, and p22phox. Furthermore, it does not require the binding of cytosolic subunits to carry out its activity. NOX4 presents an activity similar to that of superoxide dismutase (SOD) so that the superoxide that is generated is rapidly converted to H_2_O_2_ [41, 53, 83].

Lastly, there are the DUOX 1 and 2. These isoforms present a high level of expression in the thyroid gland and they are known as thyroid oxidases, although they have also been identified in the gastrointestinal tract and prostate. The main difference between DUOXs and the rest of NOXs is that they present an additional intracellular region containing two EF-hands motifs preceded by a seventh transmembrane fragment ended by an extracellular peroxidase-homology domain (PHD) in the N-terminal region, and require the presence of maturation factors (DUOXA1/2) for their activity. Like NOX4, DUOX1 and 2 generate H_2_O_2_, whose production is dependent on calcium and phosphorylation [4, 17, 29, 30, 45, 68, 105].

## NOX5

The last member of the NOX family to be identified was NOX5. In 2001, two independent research groups first detected the mRNA of a protein with high homology to NOX1 and 2. This mRNA encoded the NOX5 protein, and was detected in spleen, kidney, and testis samples. Despite the high degree of homology, NOX5 presented a great structural difference compared to the rest of NOXs, the presence of calcium-binding domains (Ca^2+^) at the N-terminus [12, 26]. From the research carried out by Cheng et al., it was determined that the human NOX5 protein constituted its own phylogenetic branch, quite different from the rest of the NOXs. This suggests that NOX5 perhaps represents the most similar protein to the ancestral NOX [26]. Due to several evolutionary studies, it has been observed that various plants, animals, and some fungi incorporate enzymes similar to NOX5, which suggests that the appearance of the first NOX5 occurred before the evolutionary separation of the kingdoms. These analyses have also served to find out that this protein has disappeared or duplicated itself in some organisms. For example, up to 10 NOX5-like genes have been detected in plants. In contrast, in organisms such as rodents its existence has not been detected [14, 63].

Based on this information, some experts refer to this oxidase as “the enigmatic NOX” [103]. The reasons that justify this name are as follows:

1. *NOX5* gene is not present in rodents.
2. It does not require the union of other subunits to be active.
3. Its structure incorporates four Ca^2+^ binding domains at the N-terminus.
4. Its activity is regulated by the intracellular concentration of Ca^2+^.
5. The activation of this oxidase requires protein conformational changes.
6. NOX5 is not N-glycosylated, in contrast to other NOXs.

## *NOX5* gene

In humans, the *NOX5* gene is located on the long arm of chromosome 15 and is composed of 18 exons. Through alternative splicing of exons, six variants have been identified: α, β, δ, γ, ε (truncated variant), and ζ. Two gene transcription start sites have been identified by studies using the 5′ RACE technique. One of them is for the β and δ variants (exon 4) and another is for the α and γ variants (exon 3). In the case of the ε isoform, its start is located in exon 6. The rest of the exons are common to all isoforms [14, 35, 97, 103].

As mentioned above, *NOX5* mRNA was initially identified in testis, spleen, and kidney samples [12, 26]. Over the years, the presence of this enzyme in other tissues and cell types has been demonstrated. This is the case of endothelial cells, vascular smooth muscle cells, and cardiomyocytes of the cardiovascular system [74, 80, 108]. Other tissues where this protein is expressed are bone marrow, uterus, stomach, skeletal muscle, and in the placenta [103]. Previous work carried out in our research group also demonstrated its expression in hepatic stellate cells [6]. Regarding the isoforms, their expression depends on the cell type analyzed. The β variant is usually the most frequent and has been detected in lung smooth muscle or podocytes. For its part, the ε variant, which was thought to be practically inactive, causes the increase in reactive oxygen species (ROS) characteristic of Barrett’s carcinoma [86, 98].

The promoter region of the gene presents alternative binding sites for different transcription factors as follows: AP-1, C/EBP, NF-κB, and STAT [75, 86]. Moreover, NOX5 gene expression can be induced by using different agonists. Angiotensin II represents an example of an agonist whose binding to its receptor in vascular smooth muscle and endothelial cells can produce an increase in the expression of NOX5 both at the mRNA and protein levels [80]. In the same work, the existence of another agonist, endothelin 1, was also confirmed. NOX5 is a protein capable of inducing various cellular changes when it binds to the endothelin receptor. One of these changes is the increase in ROS levels. Other molecules that have been described as NOX5 inducers are platelet-derived growth factor (PDGF), tumor necrosis factor-alpha (TNFα), or leptin [56, 74, 86].

Concerning the epigenetic regulation of the gene, some microRNAs (miRNAs), a class of non-coding RNAs that play important roles in regulating gene expression, have been postulated to modulate NOX5 expression. Thus, miR-15a-3p, present in circulating exosomes from diabetic patients, appears to reduce NOX5 expression within human endothelial cells [107]. On the other hand, in an in vitro model of diabetic nephropathy, miR-485 suppresses inflammation and proliferation of human mesangial cells by suppressing the expression of NOX5 [106]. Finally, the in silico analysis of miR-4321 and miR-4270, miRNAs that are upregulated in the serum of sepsis-induced acute kidney injury patients, predicts that they are able to decrease NOX5 expression [40].

## Structure and localization of NOX5

The human NOX5 protein has not yet been crystallized. However, in 2017, the structure of the ortholog of the cyanobacterium *Cylindrosperum stagnale* was determined. Due to this work, it has been possible to carry out experiments that allow studying the mechanism of O_2_^•−^ production derived from FAD domains [73].

**Fig. 2:**
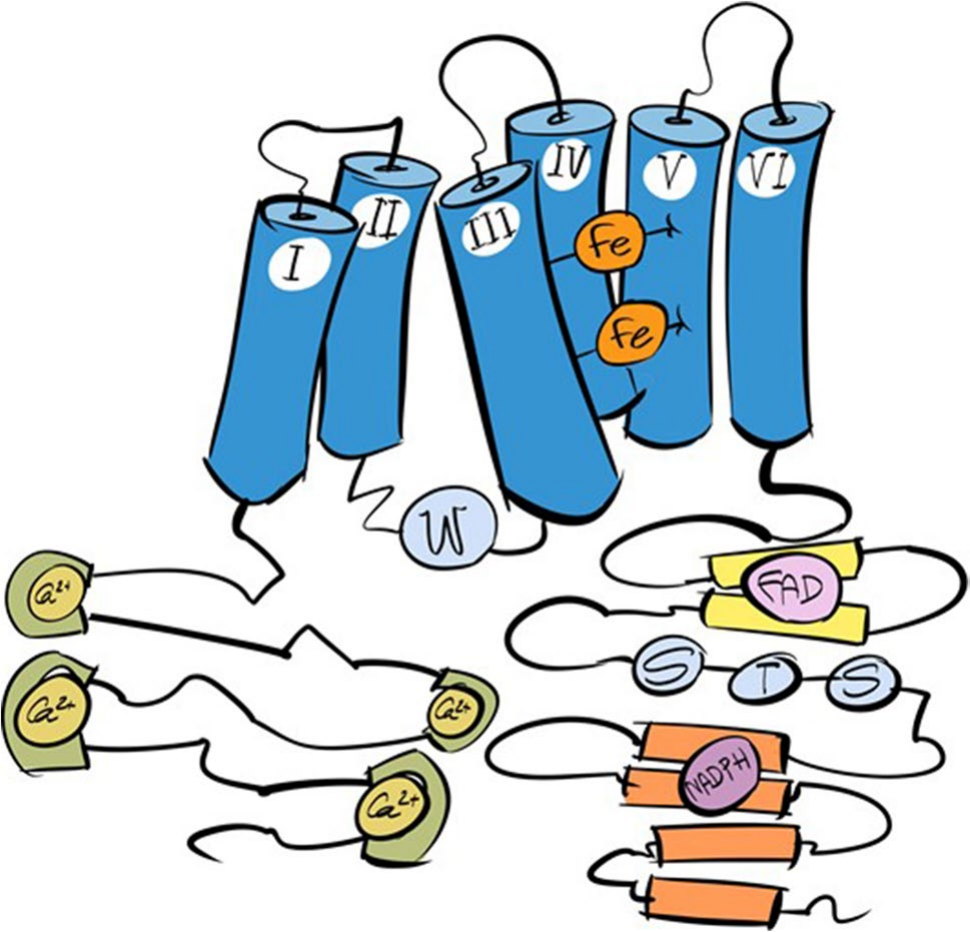
Schematic representation of the structure of NOX5. The protein contains six transmembrane domains (dark blue), with the N- and C-terminus disposed toward the cytosol. At the amino end, the EF-hand motifs are located, capable of binding Ca^2+^ ions (golden). The FAD domain (yellow) and the NADPH binding motif (purple), responsible for the production of O_2_.^•−^, and the polybasic domain (PBR-C) (pale blue) are located at the carboxyl end. Image adapted from Touyz et al. [103]

From the crystallized structure and through comparative studies with other NOXs, it has been concluded that the main components for electron transport are conserved in the NOX5 protein (Fig. 2). These are the six transmembrane domains with two histidine-linked Fe groups that link α-helices 3 and 5, the FAD-binding domain, and the NADPH-binding motif located at the C-terminus [12]. Along with these conserved elements, NOX5 includes other characteristics that influence its biochemical properties. The major difference over other enzymes of the same family is that, as mentioned above, the N-terminal end of NOX5 contains four EF-hands motifs, which allow the binding of Ca^2+^ ions. It also includes a polybasic domain (PBR-N), which is present at the carboxyl end (PBR-C). It is a region rich in residues susceptible to being phosphorylated (S and T) and with a characteristic calmodulin-binding region [13, 35].

**Fig. 3:**
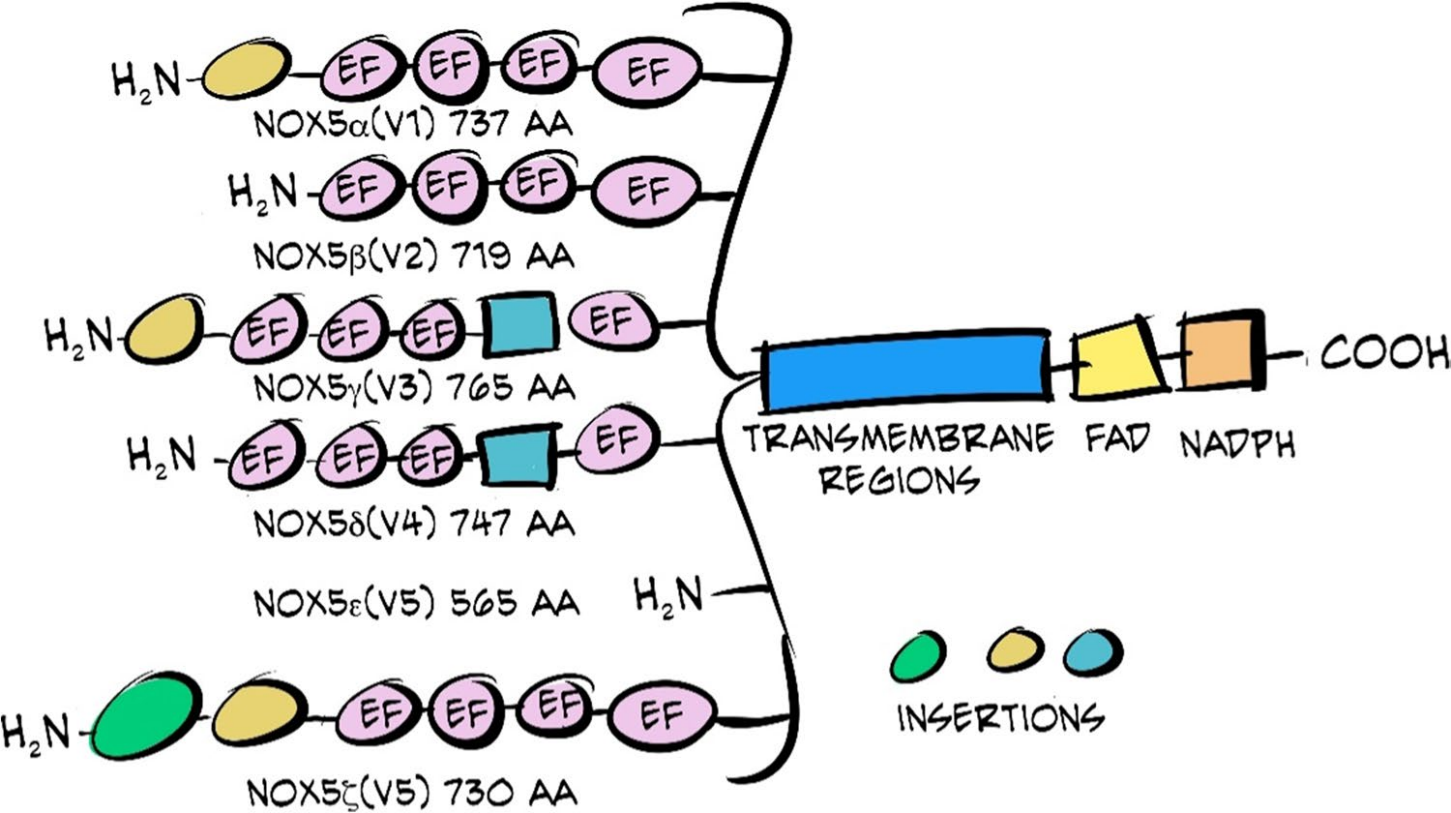
Schematic representation of the structure of the six NOX5 variants. The transmembrane domains and the C-terminus are identical for all isoforms. The main differences are present at the amino end. Image adapted from Fulton et al. [35]

In the case of the NOX5 variants, with the exception of the δ isoform, there are no major differences in their structure (Fig. 3). All NOX5 variants present six transmembrane domains and the same characteristics of the C-terminal region. However, at the amino terminus, there are slight divergences among variants. The α and β isoforms are the most active and they do not present insertions among the EF-hands motifs. In contrast, in the case of γ and δ, there is an insertion between these motifs that seems to affect the activity of the enzyme. The ε isoform, which does not seem to present much activity either, is characterized by the absence of EF-hands motifs. Finally, the ζ isoform is quite similar to the α but differs in that it hardly shows any activity [86].

Finally, in contrast to the rest of NOXs, NOX5 is mainly located in intracellular membranes such as the endoplasmic reticulum and the perinuclear area. Its presence in the plasma membrane appears to be limited to the release of ROS into the extracellular space [3, 62]. The reason why NOX5 localizes to the reticulum is not entirely clear but this might be related to the fact that it is an organelle rich in intracellular Ca^2+^, thus establishing a regulation of NOX5 via the endoplasmic reticulum [81]. The transition of NOX5 from the reticulum to the plasma membrane seems to be mediated by the carboxy-terminal PBR domain, as it is capable of binding the membrane phospholipid phosphatidylinositol 4,5-bisphosphate [62].

## Regulation of NOX5 activity

The molecular mechanism by which NOX5 generates O_2_^•−^ is the same as the one described above for NOX2. Briefly, NADPH donates electrons to the FAD domain, which binds to the heme group of the transmembrane regions. This entails the reduction of the O_2_ molecule to generate O_2_^•−^. However, the regulation of NOX5 activity presents relevant differences compared with other oxidases of the family. Although NOX5 can bind to the p22phox subunit, it has been described that such interaction is not required for NOX5 activity [91]. In fact, as observed at the time of its discovery, the activity of the enzyme depends mainly on its binding to Ca^2+^ [12, 26].

### Calcium-dependent allosteric regulation

As previously mentioned, the regulatory domain (called NOX5-EF) present in the N-terminal end of NOX5, includes four EF-hand domains that allow Ca^2+^ binding. These motifs act two by two, with those closer to the end having the lowest affinity for Ca^2+^. The binding of Ca^2+^ to these motifs induces a conformational change in the structure of NOX5 characterized by the interaction of some hydrophobic residues of the amino end with the C-terminal catalytic domain activating the enzyme. The C-terminus incorporates an autoinhibitory domain called the EF-hand binding domain. Interaction of the Ca^2+^-bound N-terminus with this region removes the inhibition, facilitating the activation of the enzyme [13, 101]. The production of ROS derived from NOX5 requires high concentrations of intracellular Ca^2+^. Nevertheless, since the enzyme is capable of producing ROS in situations where Ca^2+^ concentrations are low, there must be other mechanisms that regulate NOX5 activity.

### Regulation by covalent modification (phosphorylation) of NOX5

In 2007, Jagnandan D. et al. [54] demonstrated another form of regulation of NOX5 activity, independent of Ca^2+^. These studies found that the protein kinase C (PKC) activator compound phorbol 12-myristate 13-acetate (PMA) was able to activate NOX5 without an increase in intracellular Ca^2+^ levels. In addition, a synergy between PMA and Ca^2+^ was observed. That is, the presence of PMA caused the enzyme to be even more sensitive to the action of Ca^2+^. At the C-terminal end of the protein, there is a series of serine and threonine residues (T512 and S516) that can be phosphorylated by PKC. Mutation of these residues to alanine reduced NOX5 activation in the presence of PMA [54]. Within the PKC family, phosphorylation was found to be dependent on the α isoform as its silencing reduced the ability of PMA to activate O_2_^•−^ production [22]. Likewise, it seems that the kinases regulated by extracellular signals (ERK1/2) would also regulate the activity of NOX5 [84].

### Other types of regulation

NOX5 activity can also be regulated by other types of post-translational modifications. These changes can occur through oxidation, nitrosylation, sumoylation, and palmitoylation. The oxidation of the enzyme seems to cause a reduction in its activity since it reduces the Ca^2+^ binding capacity of the protein. Nitrosylation, together with sumoylation, also reduce its activity. Instead, palmitoylation appears to regulate the cellular localization of the enzyme [103].

Taking into account the role played by PKC in the regulation of oxidase activity, the possibility that other proteins could interact with NOX5, modifying its activity, has been evaluated. In this sense, the first protein described was calmodulin, for which NOX5 displays a binding domain at the C-terminal end and allows the oxidase to be active without the need for high Ca^2+^ levels. In turn, it has been reported that certain calmodulin inhibitors such as KN-93 are also capable of inhibiting the activity of NOX5 [85].

NOX5 also interacts with the chaperone Hsp90. This interaction results critical for the oxidase activity, since its inhibition compromises the ability of NOX5 to generate O_2_^•−^. Furthermore, inhibition of Hsp90 leads to the binding of another chaperone, Hsp70, which induces the degradation of NOX5 in the proteasome [72]. Finally, another protein that exhibits direct binding to NOX5 is caveolin 1 (Cav1). Previously, the existence of a direct union between Cav1 and NOX2 had already been described, a fact that facilitated the activation of this member of the family [71]. In the case of NOX5, in 2014, an article was published for the first time linking both proteins and reflecting the opposite of what was observed with NOX2. Cav1 negatively regulated the activity of not only NOX5, but also NOX1-3 [21]. In fact, in 2020, the presence of NOX5 and NOX1 in the caveolae of the plasma membrane in smooth muscle cells was established and the negative regulatory role that Cav1 plays on the activity of both enzymes was confirmed [5].

## Role of NOX5 in physiology and pathophysiology

### NOX5 in physiology

**Fig. 4:**
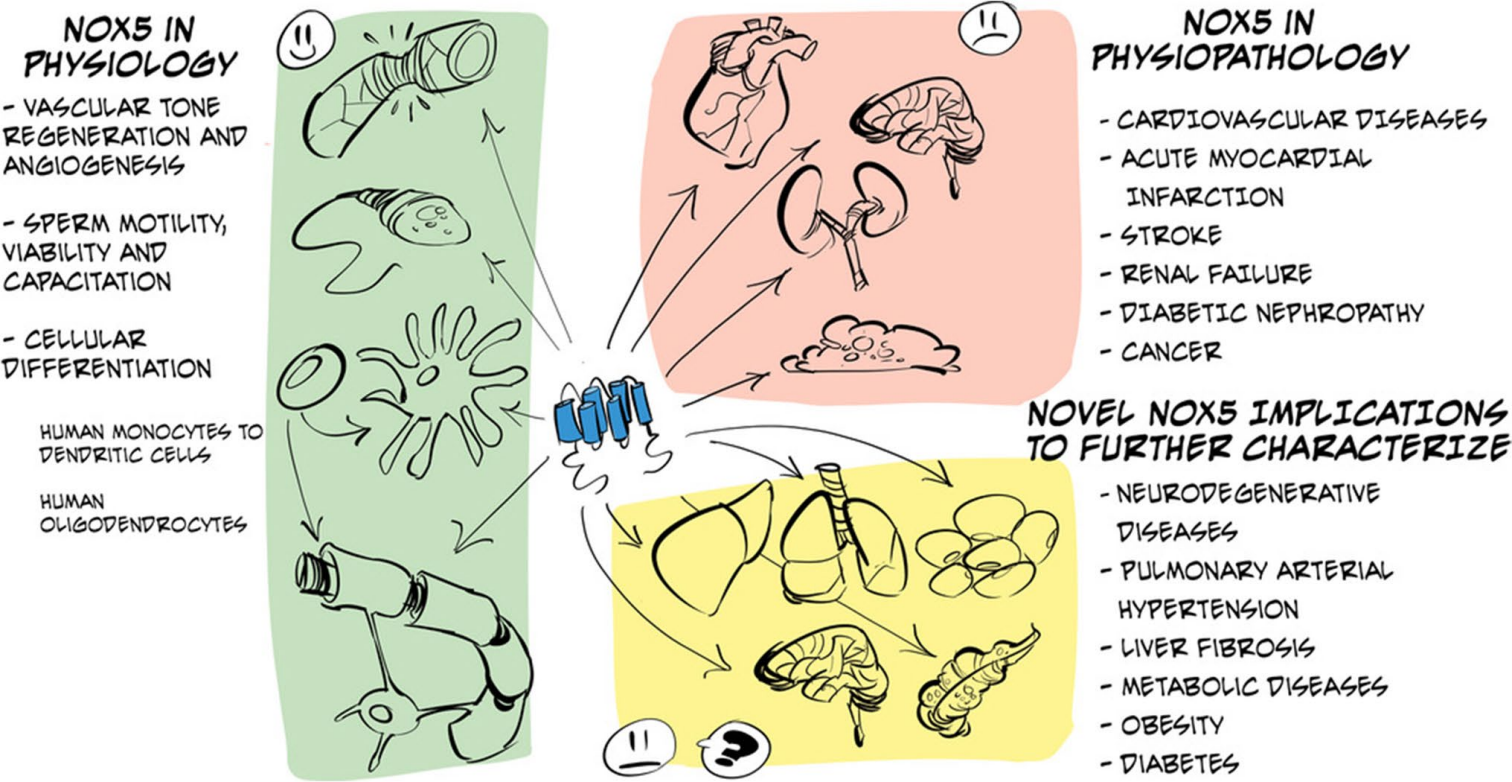
Schematic representation of the main physiological and physiopathological functions described so far for NOX5

The presence of NOX5 in different cell types and the variability in its expression make it ambiguous whether its expression promotes positive or negative effects on the tissues in which it is expressed. Its basal activity could be related to physiological effects mediated by oxidative signaling, whereas its overexpression or activation tends to cause oxidative stress-induced physiopathological damage (Fig. 4).

#### Cellular differentiation

The first studies dedicated to this protein determined that NOX5 is expressed in human monocytes and macrophages and that it is capable of regulating the differentiation of monocytes to dendritic cells [78]. However, these studies were carried out with leukemia cell lines, so the physiological effects of NOX5 on neutrophils are not entirely clear. Regarding the involvement of NOX5 in cellular differentiation, it has been confirmed that NOX5 is required for the proper differentiation of human oligodendrocytes [1].

#### Cardiovascular function

NOX5 is also expressed in other non-immune cell types and appears to modulate functions related to processes in which ROS are involved. In this sense, physiological levels of ROS are essential for normal vascular functions, including endothelial homeostasis and smooth muscle cell contraction [25]. The NOX family represents the principal enzymatic source of ROS in the vasculature [77]. Additionally, among the NOX isoforms present in human vessels, NOX5 seems to be the major ROS-generating oxidase [60, 102]. In this system, the presence of NOX5 has been confirmed at the vascular wall in endothelial cells, vascular smooth muscle cells (VSMC), and interstitial fibroblasts, as well as in blood cells themselves, such as circulating monocytes and macrophages [60]. Considering all these factors, it has been described the involvement of NOX5 activity in the physiological regulation of vascular tone [81] and angiogenesis [89]. Moreover, in relation to cardiac muscle, as previously mentioned, NOX5 is slightly expressed at the endothelium of intramyocardial blood vessels and in cardiomyocytes [48, 109], and both Ca^2+^ and ROS-mediated signaling are responsible for controlling cardiomyocyte contraction. In this context, NOX5 has been shown to have a key role in the regulation of muscle cell contraction, cell proliferation, and capillary generation [81, 89]. However, the expression of NOX5 in necrotic and non-necrotic as well as hypertrophied areas of the myocardium makes its role in the cardiovascular system difficult to interpret [48].

#### Sperm functionality

Furthermore, ROS (and more precisely O_2_^•−^) are involved in sperm functionality under physiological condition, and they are required for processes such as hyperactivation, acrosome reaction, and sperm-oocyte fusion [65]. It had been previously described that the mRNA of NOX5 is highly expressed in human testis [12], and the expression of the oxidase was identified in human [82], canine [96], and equine [95] spermatozoa. Taking all these data into account, Ghanbari et al. demonstrated that NOX5 participates in the regulation of human progesterone-activated sperm motility and viability [42]. Moreover, some studies developed in ram demonstrated that it is also involved in sperm capacitation [79].

### NOX5 in pathophysiology

On the other hand, the increase in ROS levels in the body is associated with the development of oxidative stress. For this reason, the increase in NOX5 activity is related to the progression of various pathologies such as vascular and renal diseases and cancer.

#### Cardiovascular diseases

In the case of vascular diseases, as previously mentioned, the presence and activity of the oxidase in different cell types of this system makes the circulatory system more sensitive to alterations of NOX5. Thus, human and animal studies suggest that NOX5 might promote endothelial dysfunction and inflammation in the vasculature [77]. Inflammation and oxidative stress are currently regarded as important components of atherosclerosis pathogenesis, a process in which lipid-containing plaques are formed in the blood vessel wall that can obstruct the vessel lumen or provoke erosion or rupture, inducing further thrombotic events. The implication of the NADPH family members and their role in atherosclerosis has been previously reviewed in Poznyak et al. [90]. Regarding the precise implication of NOX5 in this process, it has been described that the mRNA and protein levels of NOX5 as well as the calcium-dependent NADPH oxidase activity are significantly increased in the atherosclerotic coronary arteries from patients with coronary artery disease as compared to nonatherosclerotic vessels [47]. However, in a recent work employing a humanized Nox5 knock-in mice that expressed NOX5 in endothelial cells to test the pro-atherogenic hypothesis, Ho et al. [50] found that the expression of the oxidase per se was insufficient to induce aortic atherosclerotic lesions, even in aged mice and exposed to a high cholesterol atherogenic diet. Moreover, in the same work, the authors demonstrated that the endothelial expression of NOX5 did not aggravate aortic atherosclerosis in the atherosclerosis-prone ApoE^−/−^ mice with and without induction of diabetes [50]. Another process related to vascular diseases in which NOX5 appears to be involved is in vascular calcification, the formation of calcium phosphate crystals in the vessel wall that promotes vascular stiffness. In vitro studies employing hVSMCs demonstrated that the oxidase would be mediating the phenotypic switching between the normal “contractile VSMCs” to a dedifferentiated “synthetic VSMCs.” In addition, the work presents that in these synthetic VSMCs, Ca^2+^-NOX5-induced ROS would induce an increase in the release of extracellular vesicle (EV) as well as a decrease in their uptake, causing their extracellular accumulation and increasing calcification [36]. In the case of cardiovascular diseases, the expression of the oxidase seems to be increased in intramyocardial blood vessels and cardiomyocytes after acute myocardial infarction [48] and in the cardiomyocytes of failing human hearts [109]. Due to the regulatory role it exerts on cardiac tissue, NOX5 also seems to be involved in the development of heart attacks, hypertension, and cardiac hypertrophy [52, 64, 109]. Our group has also observed that NOX5-derived ROS may modulate the COX-2/PGE2 axis in endothelial cells, which might play a relevant role in the pathophysiology of heart infarction [76].

#### Neurological diseases

In the case of the brain, the works from the group of Harald Schmidt associate the expression of human NOX5 in endothelial and hematopoietic cells in a knock-in mouse model and in vitro organotypic hippocampal cultures, with the presence of hypertension and the risk of stroke [20, 64]. The authors show that NOX5-dependent ROS formation compromises the integrity of the blood–brain barrier, increasing infarct size and aggravating neurological function after cerebral ischemia and reperfusion injury.

#### Diabetic nephropathy

Likewise, it has been observed that NOX5 is expressed in renal tubule cells and that in conditions of diabetic nephropathy, its overexpression affects the correct functioning of the renal system. Specifically, NOX5 overexpression produces an increase in inflammatory cytokines that result in the appearance of kidney damage [52, 60]. In this sense, a recent paper of Jha et al. suggests that NOX 5 could be playing a much more prominent role than NOX4 in this disease [59].

#### Cancer

Elevated NOX5 levels have been also reported in various types of tumors and cancer cell lines (lymphoma, Barrett’s esophageal adenocarcinoma, gastric, melanoma, colon, breast, and prostate), where an increase in ROS levels correlates with increased cell proliferation, DNA damaged, angiogenesis, and reduced apoptosis [7, 9, 33, 37, 69, 93].

The activity of the oxidase has been associated with different roles in cancer progression. At the cellular level, in vitro studies have confirmed that NOX5 promotes proliferation and survival and reduced apoptosis in prostate carcinoma cancer cells, breast cancer cells, lymphoma, Barrett esophageal adenocarcinoma cells, and melanoma [7, 19, 33, 34, 51]. Using human colon and breast cancer cell lines in which the expression of NOX5 was depleted employing specific siRNAs, some works demonstrated the direct involvement of NOX5 in the migration or cell motility of those transformed cell lines [8, 33]. In addition, the activity of NOX5 is also responsible for the increased tumor cell invasion of human prostate, colon, and breast tumor cell lines [8, 33, 66]. Moreover, NOX5 could be acting as a driving oncoprotein through the regulation of the expression of several cytokines to provide the conditions that facilitates tumor malignant progression [24].

On the other hand, the clinical relevance of the activity of NOX 5 in oncology patients presenting different types of cancers has also been described. Hence, NOX5 expression was found to be increased at the invasive front of prostate cancer tumors, increasing the ROS levels in the area. The ROS produced, could potentially contribute to the expression of an active form of HIF1, that in turn, can increase the expression of the metalloproteinase 14, which was found to be also increased at the invasive front of prostate cancer tumors. NOX5 expression could be thus contributing to conferring an invasive advantage to tumor cells [66]. In the case of lymphomas, the work of Gonçalves et al. [43] demonstrated that NOX5 upregulation was associated with the acquisition of an aggressive phenotype. NOX5 expression was increased in esophageal squamous cell carcinoma (ESCC) tumors, and this elevated NOX5 was correlated to malignancy of (ESCC) tumors and poor prognosis, as a strong expression of the oxidase positively associated with advanced-stage, higher grade tumor status and higher grade lymph node status. Furthermore, those patients presenting a higher expression of NOX5 showed a shorter overall survival time. NOX5 induced the malignant progression of ESCC by activating the proto-oncogenic protein tyrosine kinase Src, particularly under hypoxic conditions [23]. NOX5 upregulation was also associated as a poor prognostic factor and worse prognosis in colon cancer patients, as the 5-year progression-free survival rate of NOX5-positive patients was significantly worse than that observed in NOX5-negative patients. Those NOX5-positive patients presented a higher local recurrence rate than that observed in NOX-5-negative patients. Besides, NOX-5 positive expression was significantly correlated with poorer differentiated histology [9].

### Roles of NOX5 to be determined

Recent studies have also suggested the involvement of NOX5 in the development of other different process in which the precise function and physiological or pathophysiological consequences of the presence and activity of the oxidase are not yet fully characterized [103]. In this context, the implication of other NADPH oxidases and oxidative stress in pulmonary hypertension or neurodegenerative disorders and neuroinflammation has been previously described, though the precise role of NOX5 in those events remains elusive [87, 100]. Related to neurological disorders, our group has recently described that the endothelial expression of NOX5 in a knock-in mouse model alters the integrity of the blood–brain barrier causing loss of memory in aged animals [27]. Other work from our group also described for the first time expression and functional relevance of NOX5 in the human cell line of hepatic stellate cells (HSC) LX-2 [6]. This cell line, when activated, presents a proliferative and myofibroblastic phenotype with increased production of collagen type I and other extracellular matrix proteins responsible for the development of liver fibrosis. The work of Andueza et al. [6] confirmed that NOX5 activity contributes to the proliferation of the cell line as well as the increased production of collagen type I, pointing to a possible implication of the oxidase in the initiation and progression of liver fibrosis, in line with the role previously reported played by NOX1,NOX2, and NOX4 [70].

#### Metabolic diseases

In relation to metabolic diseases, several works have analyzed the effect of NOX5 in diabetes. It has been described that high concentrations of glucose can increase NOX5 expression and activity [16, 22]. The activity of the oxidase tends to demonstrate a detrimental effect in this condition, mainly due to the induction of vascular complications such as diabetic nephropathy [57], but it is also related to other complications related to the disease as the presence of foot ulcers [107], vascular retinopathy [31], or the formation of abdominal aortic aneurysms [50]. In this sense, the kidneys are probably the organs in which the effect of the activity of NOX5 has been better characterized in this situation. In patients with diabetes, there is an increased expression of renal NOX5 associated with enhanced ROS formation and the upregulation of ROS-sensitive pathways as early growth response 1 (EGR-1), protein kinase C-α (PKC-α), and thioredoxin-interacting protein (TXNIP). Moreover, in animal models of diabetic kidney diseases, the overexpression of NOX5 enhanced kidney damage by increasing albuminuria, inflammation, and renal fibrosis due to the increase of ROS and the activation of the previously mentioned ROS-sensitive pathways [58]. A recent work suggested that pancreatic (β‐cell‐specific) doxycycline‐inducible expression of NOX5 in RIP/rtTA/NOX5 transgenic mice can negatively alter insulin action in high-fat diet-fed mice [16]. All these data are consistent with the idea that the expression of NOX5 tends to increase in several diseases, generally causing a worsening of the pathology [57].

However, other studies have suggested that the activity of NOX5 could present beneficial effects in other diseases, leading to an improvement in certain conditions such as cardiac remodeling or the differentiation of monocytes into dendritic cells [48, 78]. For example, disruption of the NOX5 gene employing CRISPR/Cas9 aggravates atherosclerosis in rabbits that were administered an atherogenic diet based on a high-fat cholesterol-rich (0.5% w/w) diet to induce plaque formation, suggesting a protective role for the oxidase against atherosclerosis in this model [88]. Some works from our group demonstrated that *knock-in* transgenic mice expressing endothelial NOX5, presented lower body weight gain and less mesenteric and epididymal fat mass compared to control mice. Those endothelial NOX5-expressing transgenic mice also showed significantly lower glycaemia and improved insulin-induced glucose uptake, which was accompanied by increased expression of Glut4 and Cav1 in the adipose tissue of these animals [38]. Likewise, 3T3-L1 adipocytes treated with conditioned media from NOX5-expressing endothelial cells previously incubated with high glucose and palmitic acid, presented lower lipid accumulation and higher glucose uptake. In this context, another study from our group showed that, in high-fat diet-fed endothelial NOX-5-expressing transgenic mice, endothelial NOX5 activity promotes thermogenesis and lipolysis in the mesenteric and epididymal fat through phosphorylation of STAT3 and AMPK, respectively [39]. Those effects were mediated by activating interleukin-6 (IL-6) production. Moreover, 3T3-L1 adipocytes treated with conditioned media of endothelial NOX5-expressing cells previously incubated with high glucose and palmitic acid also presented higher expression of thermogenic and lipolytic genes. All these examples illustrate the need for additional studies in order to address other potentially beneficial effects that NOX5 activity could be mediating in other diseases.

As mentioned above, in the recent years, the development of transgenic models of the expression of NOX5 in mice, has enabled to demonstrate the involvement of the oxidase in several new functions. In some of those new roles, as in the field of metabolic diseases, liver fibrosis, pulmonary hypertension, neurodegenerative disorders, or neuroinflammation, the precise effect of the activity of NOX5 has to be better characterized, since it is not still clear whether its activity is being part of the damage, or an attempt of the body to activate compensatory mechanisms to solve an insult [6, 38, 39, 87, 100]. In this sense, it would be desirable to study how the expression and activity of NOX5 are modulating the endothelial function in processes that result critical in vascular structures involved in natural barriers in the organism like the blood–brain barrier, renal glomerulus or the alveoli of the lungs. Besides, further studies should be performed in order to obtain specific inhibitors for this oxidase. In this regard, it is the first and only crystallized NADPH oxidase and there are relevant studies suggesting that NOX5 could be a good therapeutic target in vascular diseases as diabetic nephropathy [59, 67, 87], cerebral ischemic injury [18], acute myocardial infarction or stroke [77], as well as in some types of cancer [9] and a potential target of cancer cell sensitivity to chemotherapies, such as cisplatin [32, 61]. Lastly, future works would be needed to explore the epigenetic regulation of the NOX5 gene and the posttranslational regulation of the protein which are still unknown so far, and the interaction with other proteins that affects its activity.

## Conclusion

In summary, in humans, NOX5 modulates functions related to processes in which ROS participate. Although in some cases, NOX5 has been associated with the development and worsening of several pathologies and metabolic complications, such as in cardiac ischemia or pancreatic beta-cells, in other circumstances it may play a beneficial role in the condition of metabolic stress by adapting adipocyte metabolism. For example, it seems to induce a protective adipose tissue adaptation to the excess of nutrients caused by a high-fat diet in order to avoid, or at least delay, lipid accumulation and insulin resistance development. This may be mediated by an activation of the secretion of IL-6. Nonetheless, further research is needed in order to totally unveil the role of this protein in human health and disease.
